# Nonnative Audiovisual Speech Perception in Noise: Dissociable Effects of the Speaker and Listener

**DOI:** 10.1371/journal.pone.0114439

**Published:** 2014-12-04

**Authors:** Zilong Xie, Han-Gyol Yi, Bharath Chandrasekaran

**Affiliations:** 1 Department of Communication Sciences and Disorders, The University of Texas at Austin, Austin, Texas, United States of America; 2 Institute for Neuroscience, The University of Texas at Austin, Austin, Texas, United States of America; 3 Center for Perceptual Systems, The University of Texas at Austin, Austin, Texas, United States of America; 4 Cognitive Neuroscience, The University of Texas at Austin, Austin, Texas, United States of America; UNLV, United States of America

## Abstract

Nonnative speech poses a challenge to speech perception, especially in challenging listening environments. Audiovisual (AV) cues are known to improve native speech perception in noise. The extent to which AV cues benefit nonnative speech perception in noise, however, is much less well-understood. Here, we examined native American English-speaking and native Korean-speaking listeners' perception of English sentences produced by a native American English speaker and a native Korean speaker across a range of signal-to-noise ratios (SNRs;−4 to −20 dB) in audio-only and audiovisual conditions. We employed psychometric function analyses to characterize the pattern of AV benefit across SNRs. For native English speech, the largest AV benefit occurred at intermediate SNR (i.e. −12 dB); but for nonnative English speech, the largest AV benefit occurred at a higher SNR (−4 dB). The psychometric function analyses demonstrated that the AV benefit patterns were different between native and nonnative English speech. The nativeness of the listener exerted negligible effects on the AV benefit across SNRs. However, the nonnative listeners' ability to gain AV benefit in native English speech was related to their proficiency in English. These findings suggest that the native language background of both the speaker and listener clearly modulate the optimal use of AV cues in speech recognition.

## Introduction

In an increasingly global world, nonnative speech is a common occurrence. In the United States, for example, more than 35 million adults are nonnative speakers of English [1]. Speech sounds produced by nonnative speakers can deviate significantly from native norms at segmental or suprasegmental levels [2], [3]. These deviations pose a challenge to speech perception, especially in challenging listening environments such as noisy conditions [4]. The utilization of information from both auditory and visual modalities typically improves speech perception in noise, relative to auditory-only conditions [5]–[17]. While previous studies have extensively examined the role of visual cues in speech perception, the majority of them have focused on native speakers and listeners [5]–[16]. The extent to which audiovisual (AV) processing benefits nonnative speakers is thus poorly understood. The present study investigates the extent to which visual cues are used across a number of speaker-listener groups. Specifically, we examine the extent to which these cues impact the perception of nonnative speech, relative to native speech in noise. We also examine how nonnative listeners process AV cues from native and non-native speakers.

Visual cues can provide important information about vowels, diphthongs, and place of articulation for consonants [18], [19]. These visual phonetic cues can supplement phonetic information that may be distorted in auditory speech signals by noise [5], [8]. However, it should be noted that visual phonetic cues alone produce limited intelligibility, because the visemes (i.e. the units of visual speech) correspond to multiple phonemes [20], [21]. For example, Grant et al (1998) showed that sentence recognition scores ranged from 0% to 20% (mean ± *SD*: 6.5% ±5.6%) in visual-only conditions. Hence, to maximize the AV processing benefit, the presence of a critical degree of auditory phonetic information is required to bootstrap visual phonetic cues.

Native listeners can effectively incorporate visual cues to enhance recognition of speech produced by native speakers [5]–[16]. A recent study demonstrated that they can also utilize visual cues to improve recognition of speech from nonnative speakers [17]. However, relative to native speech, the amount of AV benefit was found to be reduced for nonnative speech [17]. This inefficient AV processing for nonnative speech may be related to speaker-related as well as listener-related factors. Regarding speaker-related factors, visemes from nonnative talkers may vary in their number and/or distinctiveness from native visemes norms, making nonnative visual speech cues less effective for native listeners. This idea is supported by findings from unfamiliar regional accents [22]. With respect to listener-related factors, native listeners may be biased to perceive nonnative AV speech as less reliable, i.e., exaggerate the perceived nonnativeness of the AV speech. This exaggeration may cause native listeners ignore nonnative visual cues or unable to use these cues. As a result, the effectiveness of nonnative visual cues is further reduced [17]. In this case, to maximize the AV benefit, a greater amount of auditory phonetic information may be needed to bootstrap these nonnative visual phonetic cues in comparison to native visual cues.

Similar to native listeners, nonnative listeners also use visual cues to boost the perception of speech from native speakers [23]–[25]. For example, Wang et al. (2008) demonstrated that adding visual speech information results in improved native English phoneme identification in Mandarin Chinese listeners. But relative to native listeners, the extent of AV benefit may be restricted in nonnative listeners. It has been suggested that visemes in the native speech (i.e. task language) may be different from those used in the first language of nonnative listeners [25]. In this case, nonnative listeners may show reduced sensitivity to the visual phonetic cues in the task language. In turn, they may not accurately integrate visual and auditory phonetic cues to enhance speech perception performance in the task language. With the development of expertise in the task language, nonnative listeners can become more adept at AV speech processing in the task language [23]. Indeed, Wang et al (2008) revealed that longer exposure to English in Mandarin listeners was associated with better identification of native English phoneme in AV conditions. This association was particularly evident for phonemes that are nonexistent in Mandarin. These findings suggest the potential role of linguistic expertise in modulating the extent of AV benefit in nonnative listeners.

For the various nonnative speech perception scenarios we have discussed thus far, less effective use of AV cues can be partially attributed to the fact that the native phonemic inventories between the speaker and the listener are different. A natural question to ask is whether AV processing in nonnative speakers would be enhanced if the speaker and listener are matched in their native phonemic inventories. The acoustic-phonetic features as well as visual features of the task speech (i.e., the corpus used for speech perception tasks) produced by nonnative speakers may deviate from the norms of task speech. In the nonnative group, the shared first language knowledge (i.e. same native phonemic inventories) and second language (i.e. task language) learning experience may compensate their ability to accurately decipher these features [26]. These compensatory processes may counteract the adverse effects caused by their inadequate linguistic expertise in the task language. To our knowledge, no study has systematically examined this particular question.

For native speech perception, the amount of AV benefit critically depends on the signal-to-noise ratio (SNR) between the auditory speech signals and the background noise [9], [11]–[13]. In some studies, SNRs are chosen adaptively for individual listeners and/or for particular speech stimuli to ensure that the listeners can gain maximal AV benefit. The value is often determined as the SNR at which auditory-only performance is close to 50% correct [e.g., 14]. More precisely, a number of studies have demonstrated that the AV benefit tends to be largest at the intermediate SNR, and decreases for higher and lower SNRs [9], [11]–[13]. With respect to nonnative speech perception, existing research on AV benefit have typically used a single SNR [17], [23]. The manner in which AV benefit varies with SNR for speech perception in noise in nonnative speakers has not been examined thus far. As discussed before, nonnative speakers may demonstrate reduced ability in AV processing in comparison to native speakers. Hence, it can be expected that the manner in which AV benefit varies with SNR may be different between nonnative and native speakers.

**Figure 1:**
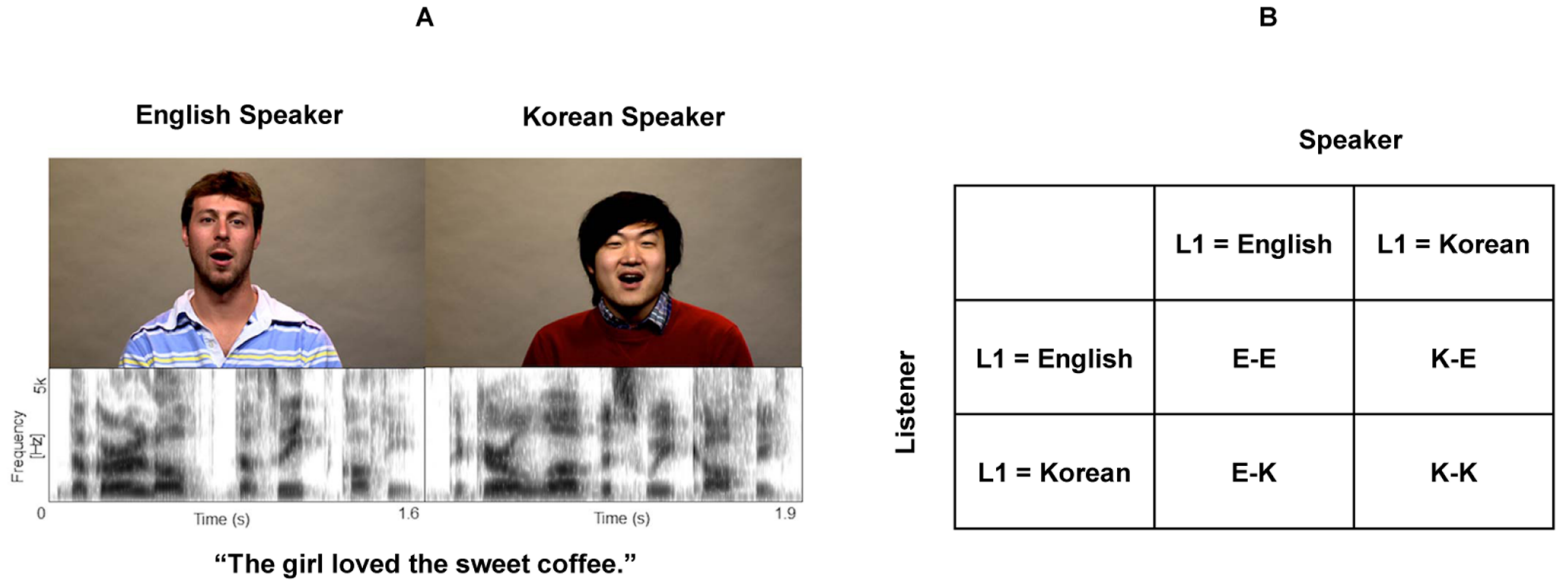
Stimuli and the four speaker-listener groups in the current study. (A) An example stimulus. Visual (upper panel) and auditory (lower panel) speech cues of the sentence “*The girl loved the sweet coffee*” produced by a native American English speaker and a native Korean speaker. (B) The four speaker-listener groups. E-E: English sentences were produced by a native American English speaker, and then presented to a group of native American English listeners. E-K: English sentences were produced by a native American English speaker, and then presented to a group of native Korean listeners. K-E: English sentences were produced by a native Korean speaker, and then presented to a group of native American English listeners. K-K: English sentences were produced by a native Korean speaker, and then presented to a group of native Korean listeners.

The present study examines the role of visual cues in enhancing speech perception in noise across various speaker-listener pairs (native speaker and listener, nonnative speaker and listener, native speaker and nonnative listener, and nonnative speaker and native listener). Particularly, we are interested in understanding the manner in which the AV benefit is modulated by SNR, and whether the typical pattern (maximum benefit at intermediate SNRs) is present for conditions that do not involve a native-speaker and native-listener pair. This will allow us to evaluate the extent to which the typical pattern of enhanced AV processing at intermediate SNRs is influenced by native language experience. Finally, we assess the effects of linguistic expertise, based on English proficiency measures, on the AV benefit in nonnative listeners. To this end, we examined the perception of English sentences in noise across a range of SNRs (−4 to −20 dB; −4 dB steps) in the following four speaker-listener groups: E-E, E-K, K-E, and K-K (see Figure 1 for detailed descriptions of the four groups). Based on previous studies, we expected that the amount of overall AV benefit will be reduced in conditions with nonnative speakers, relative to the E-E group. Further, as with previous work, we predicted that higher English proficiency in nonnative listeners is related to larger AV benefit for native English speech [23].

## Methods

### Participants

Two groups of young adults (age range: 18–29, mean age  = 21.73) participated in the experiment. Native American English (*n* = 15) and native Korean (*n* = 15) speakers were recruited from the University of Texas at Austin community. All participants completed an abbreviated version of the LEAP-Q language history questionnaire [27]. All native Korean participants reported an English language proficiency score of 5 or higher on the language questionnaire (1: very low; 10: perfect). All participants reported normal or corrected-to-normal vision, and no previous history of language or hearing problems. Each participant underwent a hearing screening to ensure pure-tone thresholds of ≤25 dB HL at 500 Hz, 1000 Hz, 2000 Hz and 4000 Hz. Participants provided written informed consent and received monetary compensation for their participation. All materials and procedures were approved by the Institutional Review Board at the University of Texas at Austin.

### Materials

The target stimuli consisted of 80 meaningful sentences taken from the Basic English Lexicon [28]. Each sentence contained four keywords for intelligibility scoring (e.g., *The gray mouse ate the cheese*). One native male American English speaker and one native male Korean speaker were recorded producing the full set of 80 meaningful sentences.

The speakers were video-recorded on a sound-attenuated stage at The University of Texas at Austin. The video recording was captured using a Sony PMW-EX3 studio camera with target sentences presented to the speaker on a teleprompter. Camera output was processed through a Ross crosspoint video switcher and recorded on an AJA Pro video recorder. Audio was recorded with an Audio Technica AT835b shotgun microphone placed on a floor stand in front of the speaker. The speakers were instructed to speak in a conversational style, as if they were talking to someone familiar.

A 10 seconds masker track of pink noise was created using the Noise Generator option in Audacity [29]. The pink noise track was equated for root-mean-squared (RMS) amplitude to 54 dB, 58 dB, 62 dB, 66 dB and 70 dB using Praat [30] to create five 10 seconds masker tracks. Each of the five masker tracks was segmented using Praat [30] to create 80 noise clips. Each noise clip was one second longer in duration than its accompanying target sentence.

All target sentences were segmented from the long video recordings using Final Cut Pro. Forty unique sentences were selected per speaker to remove sentences with production errors. The audio was detached from each segmented video and RMS amplitude equalized to 50 dB SPL using Praat [30]. Each audio clip was mixed with five corresponding pink noise clips to create five stimuli of the same target sentence with the following SNRs: −4 dB, −8 dB, −12 dB, −16 dB, and −20 dB. For each stimulus, the noise began 500 ms before the onset of the target sentence and ended 500 ms after the target sentence's offset. The mixed audio clips served as the stimuli for the audio-only condition. The mixed audio clips were then reattached to the corresponding videos to create the stimuli for the AV condition. A freeze frame of the speaker was captured and displayed during the 500 ms noises that were before the onset of target sentence and after the offset of target sentence. In total, for each speaker, there were 200 final audio files (40 Sentences ×5 SNRs) and 200 corresponding AV files (40 Sentences ×5 SNRs).

### Design and Procedure

The study was administered in a sound-attenuated room using E-Prime 2.0 software [31]. The sound stimuli were bilaterally presented to participants over Sennheiser HD-280 Pro headphones.

There were three within-subject variables: (1) speaker: English sentences produced by a native American English speaker or a native Korean speaker, (2) presentation modality: audio-only (AO) or audiovisual (AV), and (3) SNR: −4 dB, −8 dB, −12 dB, −16 dB, or −20 dB. Four trials, i.e., four target sentences, were used in each condition. In total, there were 40 sentences produced by the native American English speaker, and 40 sentences produced by the native Korean speaker. The 80 trials were mixed and then presented to both groups of participants.

Before the experiment, participants were informed that they would listen to sentences mixed with noise and each sentence would either be audio-only or accompanied by a video of the speaker. Additionally, each participant was informed that the target sentences would always begin a half a second after the onset of the noise. In each trial, the participants initiated the presentation of the stimuli by pressing a designated key on a keyboard, and were asked to type the target sentence after stimuli presentation. If participants were unable to understand the entire target sentence, they were asked to report any intelligible words or to guess. If they did not understand any words, they were asked to type ‘X’. For trials in the audio-only condition, a centered black crosshair on a white background was presented on the screen along with the sound stimulus; for trials in the audiovisual condition, a full-screen video of the speaker was presented along with the sound stimulus.

After the experiment, each sentence was scored by the number of keywords correctly identified (4 per sentence) for a total of 16 keywords per condition per listener. Responses were scored per accurately typed keyword. Responses that included homophones and phonetic misspellings were scored as correct.

### Data Analysis

#### Speech intelligibility

The intelligibility data was analyzed with a generalized linear mixed effects logistic regression where keyword identification (correct vs. incorrect) was the dichotomous dependent variable. In the model, fixed effects included SNR, modality, speaker, listener group, and their interactions. Two alternative random effects structures were considered: (1) by-subject and by-sentence intercepts, and (2) by-sentence intercept and by-subject sentence slope. The second model failed to converge after 10,000 iterations, thus the first model was used. Original SNR values (−4, −8, −12, −16, and −20) was mean-centered, and the corresponding mean-centered values were 8, 4, 0, −4 and −8. This mean-centered SNR was treated as a continuous variable. Modality (AO or AV), speaker (American English or Korean), and listener group (American English or Korean) were treated as categorical variables. In the model, the reference levels were AO, the American English speaker and the American English listener group. To reduce the risk of overfitting the data, we systematically removed the insignificant fixed effects, and compared each simpler model to the more complex model using the likelihood ratio [32]. Only the results from the simplest, best-fitting model were reported in the results section. Analysis was performed using the lme4 1.1–2 package in R 3.0.2 [33].

#### AV benefit over SNRs

**Figure 2:**
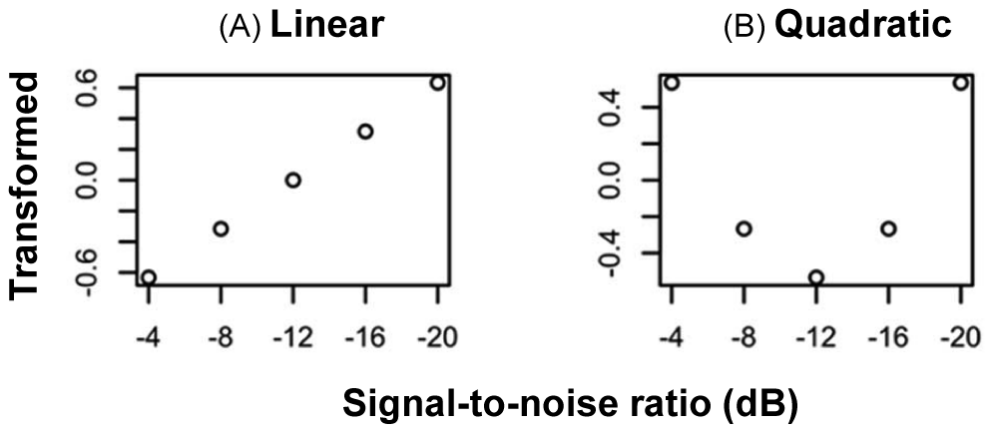
Signal-to-noise ratio (SNR) values were transformed to be used as independent variables in the linear mixed effects modeling analysis in order to test specific patterns regarding the relationship between visual enhancement (VE) and SNR. (A) Mean-centered and scaled raw SNR values. This variable presupposes a linear relationship between VE and SNR, which was used to test the pattern that the AV benefit varies linearly with SNRs, and is greatest at the highest SNRs or at the lowest SNRs. (B) The second-degree polynomial of the linear SNR values. This variable presupposes a quadratic relationship between VE and SNR, which was used to test the pattern that the AV benefit peak at intermediate SNR of −12, and drop for SNRs above and below.

For each participant, at each SNR, visual enhancement (VE) was calculated as the difference in proportion of correctly identified keywords between the AV and AO condition, using the formula: *VE = AV-AO* [12]. Note that we had also calculated VE using the formula: (AV-AO)/(2-AO), and the results were qualitatively and quantitatively similar to those with the formula: (AV-AO). We decided to use the formula (AV-AO) to be consistent with Ross, Saint-Amour, Leavitt, Javitt et al. (2007). This index quantified the amount of AV benefit in speech intelligibility at each SNR. We employed psychometric function analysis to fit the AV benefit data with two types of functions of SNR. The first was a quadratic function, which was used to test the pattern of AV benefit that peaks at intermediate SNR of −12 dB, and drops for SNRs above and below [9], [11]–[13]. This can be used to model the AV benefit pattern in native speakers. The second was a simple linear function, which supposed that the AV benefit varies linearly with SNRs, and is greatest at the highest SNRs or at the lowest SNRs. This is presented as an alternative pattern to that found in native speakers. We tested these two patterns in the four speaker-listener groups (i.e., E-E, E-K, K-E, and K-K) separately. Specifically, VE scores were analyzed using linear mixed effects modeling [33]. Individual VE scores were entered as the dependent variable. For fixed effects, 1st and 2nd degree polynomials of the mean-centered SNR values were entered to test whether the relationship between SNR and VE was linear (Figure 2A) or quadratic (Figure 2B). Random effects included by-subject intercept.

#### AV benefit and linguistic expertise

To assess the effects of linguistic expertise on AV benefit in nonnative listeners, we examined the relationship between English proficiency and the amount of AV benefit in native Korean listeners. The proficiency measures were correlated with VE scores for speech produced by the native American English speaker and the native Korean speaker separately. According to results from the above analyses, the effects of the linguistic expertise of the listeners appeared to be largest at −12 dB, hence, our analysis was restricted to this SNR.

## Results

### 

#### Speech intelligibility

**Figure 3:**
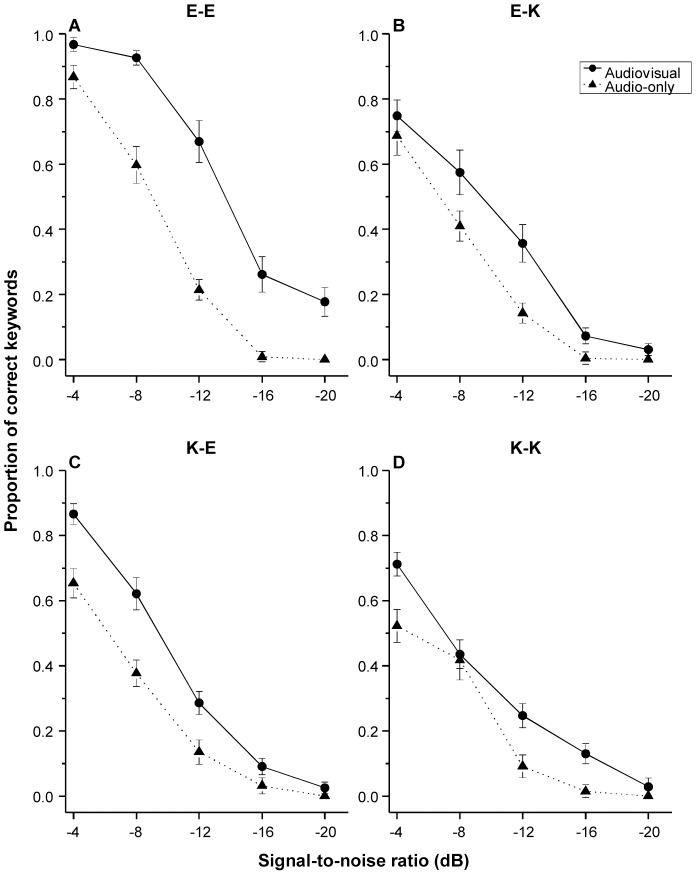
Mean proportion of correctly identified keywords as a function of signal-to-noise ratio in audio-only and audiovisual conditions across the four speaker-listener groups (see **Figure 1** for detailed descriptions of the four speaker-listener groups). Error bars represent standard error.

Figure 3 shows mean proportion of correctly identified keywords as a function of SNR in both AO and AV conditions across the four speaker-listener conditions.

Table 1 displays the estimates, *SE*, z and p values of the model results. The effect of SNR was significant (*p*<.001), where improving the SNR increased the probability of the correct keyword identification. The probability of the correct keyword identification was significantly higher for AV condition than AO condition (*p*<.001). The probability of the correct keyword identification was significantly higher for speech produced by the native American English speaker than that by the native Korean speaker (*p*<.001), The probability of the correct keyword identification was significantly higher for native American English listeners than native Korean listeners (*p* = .003). SNR interacted significantly with the other three fixed effects (i.e., modality, *p*<.001; speaker, *p* = .032; listener group, *p*<.001). These results suggest that intelligibility benefit from SNR increment was less for AV condition (relative to the AO condition), for speech from the native Korean speaker (relative to speech from the native American English), and for native Korean listeners (relative to native American English listeners). Modality also interacted with speaker (*p*<.001) and with listener group (*p*<.001). These results suggest that intelligibility benefit from visual cues was less for speech from the native Korean speaker (relative to speech from the native American English speaker), and for native Korean listeners (relative to native American English listeners). Further, the speaker by listener group interaction was significant (*p* = .006), where native American English listeners outperformed native Korean listeners, but their intelligibility performance difference was less for speech from the native Korean speaker than for speech from the native American English speaker.

**Table 1:** Results of the linear mixed effects logistic regression on intelligibility data in Experiment 1.

*Fixed effects*	*Estimate*	*SE*	*z value*	*p*
(Intercept)	−1.68	0.19	−9.06	<.001
SNR	0.48	0.02	28.21	<.001
Modality_AV	2.55	0.15	17.07	<.001
Speaker_Korean	−0.77	0.20	−3.82	<.001
Listener group_Korean	−0.62	0.21	−2.95	.003
SNR:Modality_AV	−0.10	0.02	−6.52	<.001
SNR:Speaker_Korean	−0.03	0.02	−2.15	.032
SNR:Listener group_Korean	−0.07	0.02	−4.67	.029
Modality_AV:Speaker_Korean	−0.87	0.20	−4.48	<.001
Modality_AV:Listener group_Korean	−1.26	0.20	−6.43	<.001
Speaker_Korean:Listener group_Korean	0.53	0.19	2.76	.006
Modality_AV:Speaker_Korean:Listener group_Korean	0.99	0.26	3.88	<.001

**Figure 4:**
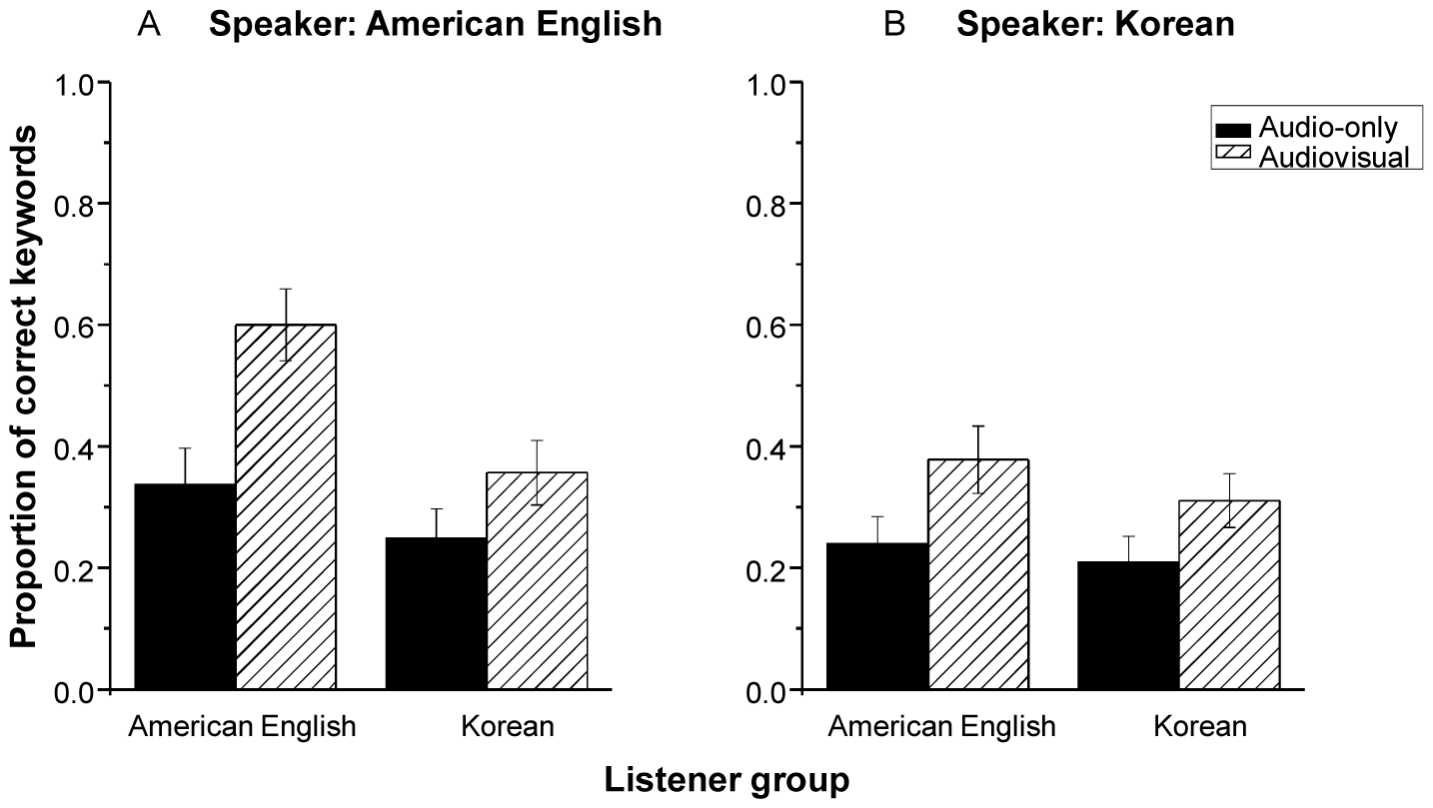
Mean proportion of correctly identified keywords as a function of modality in native American English listeners and native Korean listeners, collapsed across five signal-to-noise ratios. Sentences were produced by a native American English speaker (A) and a native Korean speaker (B). Error bars represent standard errors.

Finally, as demonstrated in Table 1, the three-way interaction among modality, speaker, and listener group was significant (*p*<.001). To understand the nature of this three-way interaction, a second round of mixed effects logistic regressions was performed for speech produced by the native American English speaker and the native Korean speaker individually. In both models, modality, listener group, and their interactions were include as fixed effects, and by-subject and by-sentence intercepts were included as random factors. Modality and listener group were treated as categorical variables. The reference levels were AO and American English listener group. As shown in Table 2, for speech produced by the native American English speaker, the modality by listener group interaction was significant (*p*<.001). The nature of this interaction was examined with multiple comparisons of Tukey contrasts. As shown in Figure 4A, although AV processing increased the probability of the correct keyword identification in both listener groups, the AV gain was less in native Korean listeners (β = 0.55, *SE* = 0.10, *z* = 5.78, *p*<.001) relative to native American English listeners (β = 1.26, *SE* = 0.09, *z* = 13.98, *p*<.001). However, for speech produced by the native Korean speaker, the modality by listener group interaction was not significant (*p* = .052). This suggests that the AV gain to intelligibility is comparable between native American English and native Korean listeners (see Figure 4B).

**Table 2:** Results of the linear mixed effects logistic regression on intelligibility data to examine three-way interaction among modality, speaker, and listener group in Experiment 1.

*Speaker*	*Fixed effects*	*Estimate*	*SE*	*z value*	*p*
American English	Intercept	−0.84	0.13	−6.52	<.001
	Modality_AV	1.26	0.09	13.98	<.001
	Listener group_Korean	−0.43	0.15	−2.75	.006
	Modality_AV:Listener group_Korean	−0.70	0.13	−5.37	<.001
					
Korean	Intercept	−1.09	0.12	−8.79	<.001
	Modality_AV	0.57	0.09	6.06	<.001
	Listener group_Korean	−0.39	0.14	−2.83	.005
	Modality_AV:Listener group_Korean	0.26	0.14	1.92	.052

#### AV benefit over SNRs

**Figure 5:**
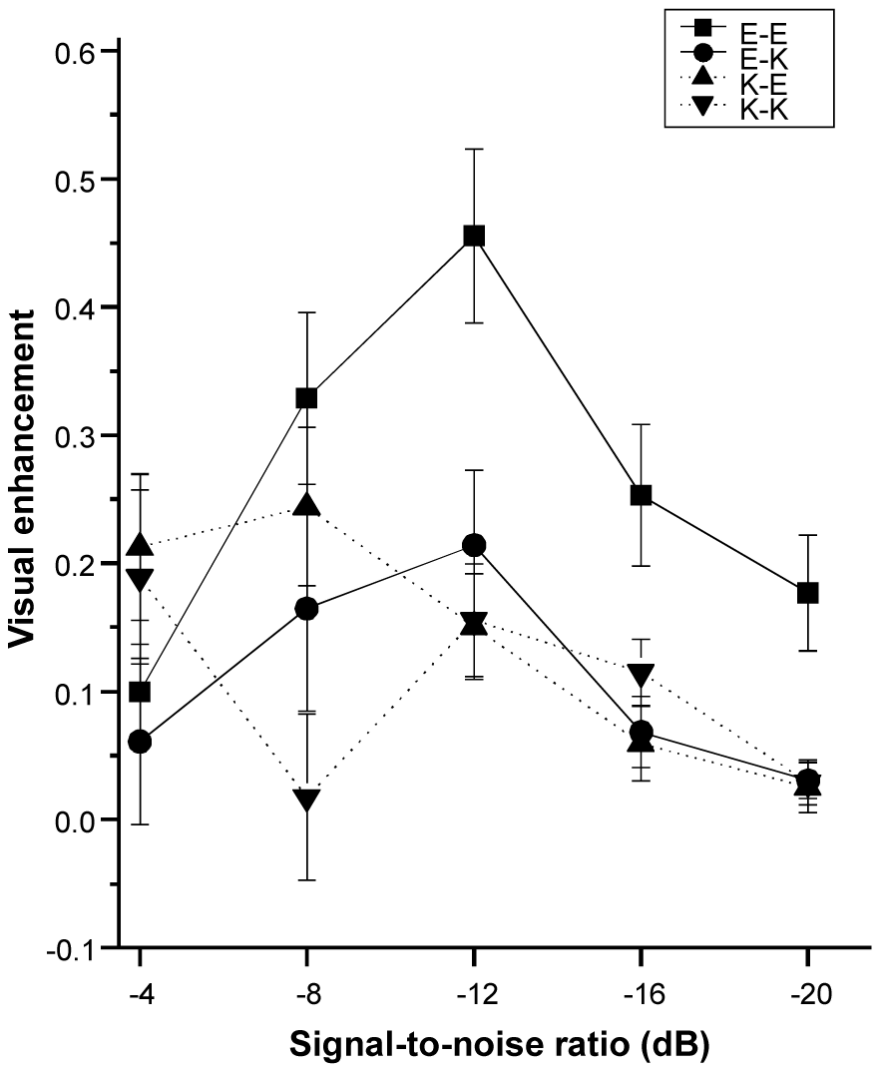
Mean visual enhancement scores as a function of signal-to-noise ratio in the four speaker-listener groups (see **Figure 1** for detailed descriptions of the four speaker-listener groups). Error bars represent standard errors.

We employed psychometric function analysis to quantify the pattern of VE as a function of SNR. As shown in Table 3, in the E-E group, the effect of first-degree polynomial of SNR was not significant (*p* = .658), presenting no evidence that VE varied as a linear function of SNR. However, the effect of second-degree polynomial of SNR was significant (*p*<.001), indicating that there was a negative quadratic relationship between SNR and VE, and the peak was at −12 dB (see Figure 5). A similar trend was also observed in the E-K group, where the effect of first-degree polynomial of SNR was not significant (*p* = .371), but the effect of second-degree polynomial of SNR was significant (*p* = .023) (see Figure 5).

**Table 3:** Psychometric function analysis on visual enhancement data in Experiment 1.

*Speaker-listener group*	*Fixed effects*	*Estimate*	*SE*	*t value*	*p*
E-E	Intercept	0.26	**0.03**	**8.16**	<.001
	first-degree polynomial of SNR	**−0.10**	**0.22**	**−0.45**	**.658**
	second-degree polynomial of SNR	**−0.97**	**0.22**	**−4.49**	<.001
					
E-K	Intercept	**0.11**	**0.02**	**4.37**	<.001
	first-degree polynomial of SNR	**0.19**	**0.21**	**0.90**	**.371**
	second-degree polynomial of SNR	**−0.50**	**0.21**	**−2.32**	**.023**
					
K-E	Intercept	**0.14**	**0.02**	**7.10**	<.001
	first-degree polynomial of SNR	**0.69**	**0.17**	**4.06**	<.001
	second-degree polynomial of SNR	**−0.13**	**0.17**	**−0.79**	**.430**
					
K-K	Intercept	**0.10**	**0.02**	**4.67**	<.001
	first-degree polynomial of SNR	**0.27**	**0.19**	**1.45**	**.151**
	second-degree polynomial of SNR	**−0.01**	**0.19**	**−0.04**	**.968**

In the K-E group, the first-degree polynomial of SNR was significant (*p*<.001), indicating that VE linearly increased as SNR increases. However, the second-degree polynomial of SNR was not significant (*p* = .430), presenting no evidence that there was a quadratic relationship between SNR and VE (see Figure 5). In the K-K group, neither the first-degree or second-degree polynomial of SNR was significant (*p* values: .151 and .968 respectively). This suggests that the AV benefit pattern in this group may be accounted for psychometric functions other than the two presented in this study (see Figure 5).

#### AV benefit and Linguistic expertise

**Figure 6:**
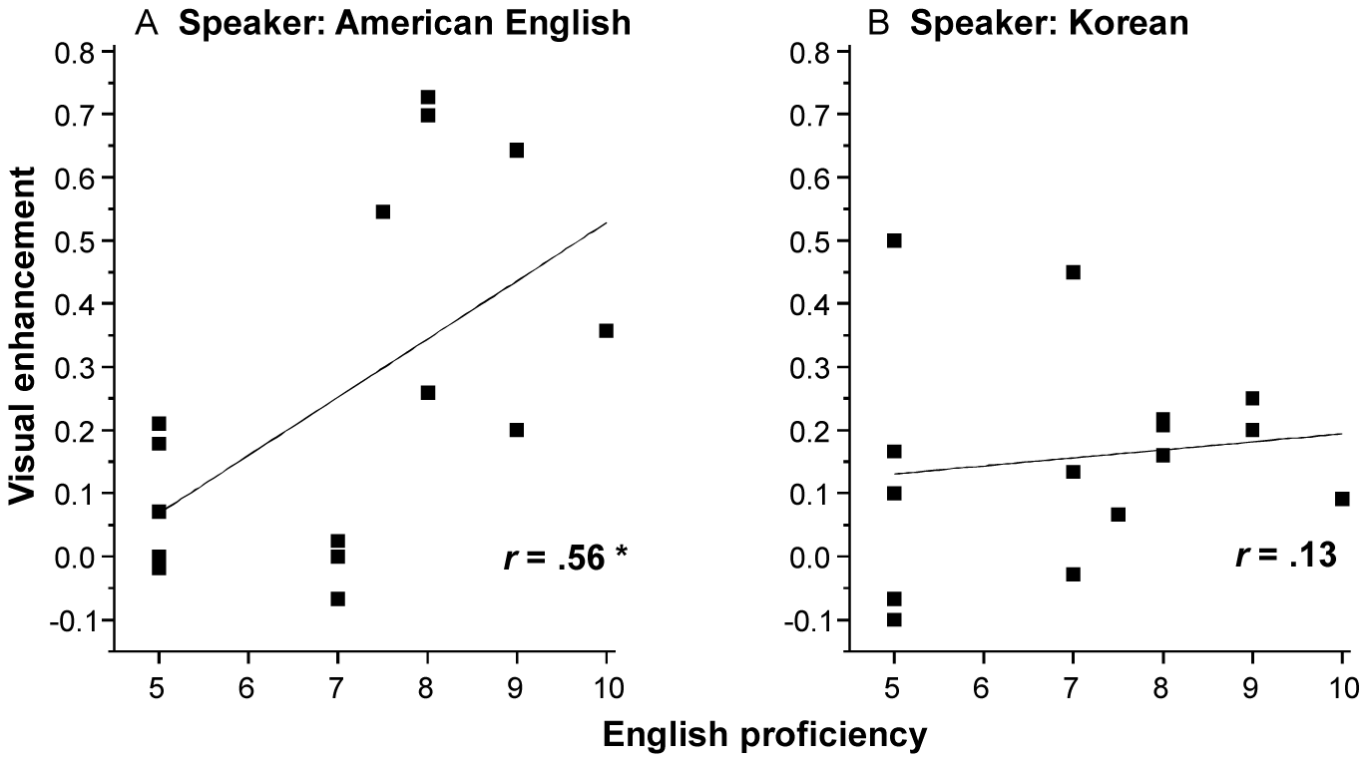
Visual enhancement scores as a function of self-reported English proficiency in native Korean listeners. (A) Sentences were produced by a native American English speaker. (B) Sentences were produced by a native Korean **speaker**. The straight line represents the best-fitting line for the data points. *r* represents the correlation coefficient between visual enhancement scores and English proficiency. * denotes *p*<.05.

Finally, we examine the impact of linguistic expertise on AV benefit to speech intelligibility in native Korean listeners. For speech produced by the native American speaker, English proficiency measures were positively correlated with VE scores, *r*(13) = .56, *p* = .03. That is, where native Korean listeners reported higher proficiency in English, a greater AV benefit was found (see Figure 6A). However, for speech produced by the native Korean speaker, English proficiency measures were not significantly correlated with VE scores, *r*(13) = 0.13, *p* = .60 (see Figure 6B).

## Discussion

The present study examined the role of visual cues in enhancing speech perception in noise across four speaker-listener groups: native speaker and listener (E-E), native speaker and nonnative listener (E-K), nonnative speaker and native listener (K-E), and nonnative speaker and listener (K-K). In particular, we examined the manner in which the AV benefit was modulated by SNR, and the extent to which the pattern found in conditions with a native-speaker and native-listener pair (enhanced AV processing at intermediate SNRs) was present for other three speaker-listener groups. Finally, we investigated the influence of linguistic expertise on the AV benefit in nonnative listeners. To this end, we examined native American English and native Korean listeners' perception of English sentences produced by a native American English and a native Korean talker in noise from −4 to −20 dB (−4 dB steps). We also collected English language proficiency data from the native Korean listeners as an index of linguistic expertise.

Consistent with our prediction, for native English speech, native Korean listeners obtained less amount of AV benefit relative to native American English listeners. Nonetheless, when perceiving nonnative English speech, both groups of listeners gained comparable amount of AV benefit. These novel findings suggest that the native language background of the speaker and listener interact to modulate AV benefit to intelligibility. Specifically, the nonnative speaker condition and the nonnative listener condition leads to diminished AV benefit to speech intelligibility respectively. But their adverse effects were not cumulative, as the K-K group did not yield a further reduction in AV benefit.

These results expand our understanding of nonnative AV speech perception, and suggest that a match in native language background between speakers and listeners may ameliorate the difficulty of face-to-face L2 communication in noise for nonnative speakers. This findings are in in concert with the “interlanguage speech intelligibility benefit” found for speech perception in auditory-only conditions, that is, for nonnative listeners, speech from a high proficiency L1 matched nonnative speaker is as intelligible as those from the native speakers [e.g., 26]. A plausible explanation for this AV processing benefit from matched native language between speakers and listeners is related to their shared first language knowledge and shared second language learning experience. These shared knowledge and experience may result in similar representation of acoustic-phonetic features and visual features for the L2 speech between these nonnative listeners and nonnative speakers. This would mitigate the detrimental effects from the inadequate linguistic expertise in nonnative listeners, in turn aid AV (and AO) speech perception in nonnative listeners in adverse listening conditions.

We employed psychometric function analysis to characterize the AV benefit pattern over SNRs across the four speaker-listener conditions. The results revealed that, regardless of the nativeness of the listeners, for speech from the native American English speaker (E-E and E-K groups), there was a negative quadratic relationship between visual enhancement (VE, i.e., intelligibility improvement in AV versus AO) and SNR, with maximal AV benefit at the intermediate SNR of −12 dB. These results accord with previous literature with native English speakers and listeners, which showed that maximal AV benefit to intelligibility occurs at intermediate SNR [9], [11]–[13]. In contrast, in the K-E group, AV benefit decreased as SNR dropped, and the SNR for maximal AV gain shifted to a higher level (−4 dB). This pattern is distinct to the pattern found for native English speech. Further, in the K-K group, the pattern did not fit with that observed for the conditions with native English speech. However, numerically, AV benefit in this instance was largest at −4 dB (see Figure 5), which is consistent with the K-E group. Taken together, these results showed that, for native English speech, the largest AV benefit occurred at intermediate SNR (i.e. −12 dB); but for nonnative English speech, the largest AV benefit occurred at a higher SNR (−4 dB).

These distinct AV benefit patterns across the four speaker-listener groups (see Figure 5) suggest that the native language experience of the speaker modulates the manner in which AV benefit varies with SNR. One factor that may partially account for these effects is the informativeness of visual cues from the speaker [17]. Compared with native visual articulatory speech cues, nonnative visual speech cues may be less informative to native listeners. While the presence of a critical degree of auditory phonetic information to bootstrap visual phonetic cues is crucial to maximize AV benefit [9], [11]–[13], the reliance on auditory speech may be more pronounced in the case of nonnative speech. This hypothesis is consistent with the findings that, for nonnative listeners, clear speech signal (i.e., there was sufficient acoustic details) is required for contextual cues to facilitating their speech recognition in noise [34]. Therefore, nonnative visual and auditory phonetic information would be optimally integrated only at relatively high SNR (i.e., -4 dB in this study, when the auditory speech is rather well retained) to maximize AV gain to intelligibility. Future studies should further investigate this assumption and delineate the mechanisms underlying the distinct patterns found between speech produced by native and nonnative English talkers.

As opposed to the nativeness of the talker, the native language background of the listener exerts negligible impact on the pattern of AV benefit over SNR. However, it does affect the extent of AV benefit that listeners can attain. This argument is further corroborated by the findings that the linguistic expertise of the native Korean listeners is related to their AV benefit in the perception of English speech produced by the native American English speaker. For example, Ross et al (2011) found that children aged 5 to 10 years old demonstrated less AV gain relative to adults, and this finding was interpreted as a result of prolonged maturation of brain regions crucial to AV speech processing until late childhood. Based on findings from the current study, we may argue that expertise, and not necessarily maturational processes may drive the ability to effectively use visual cues in a language.

## Conclusions

The present study demonstrates partially dissociable effects of the native language background of the speaker and the listener on AV processing in speech perception in noise. Specifically, while both the native language background of the speaker and listener modulated the amount of AV benefit, only the native language background of the speaker affects the relationship between AV benefit and the intelligibility of speech sounds. Further studies are needed to more precisely delineate the mechanisms underlying their distinct impact on nonnative AV speech perception. Finally, it should be noted that only two speakers (i.e., a native American English speaker and a native Korean speaker) were recruited in this study to produce the stimuli. There is the possibility that the observed effects here are specific to these individual speakers. Hence, future studies are needed to replicate our findings with more speakers or with speakers of native language other than English and Korean.
